# The Beat

**DOI:** 10.1289/ehp.121-a151

**Published:** 2013-05-01

**Authors:** Erin E. Dooley

**Affiliations:** Erin E. Dooley, MA, is a staff writer for *EHP*.

## Nickel-Tainted Flowers Taste Bad to Bees

Metals can accumulate in flowering plants grown in contaminated soils and have been found in honey samples collected near polluted sites. Investigators at the University of Pittsburgh now report that metal contamination of flowers may affect bumblebees’ foraging behavior and potentially harm pollinator health.^1^ In the study, bees spent 75% less time foraging on nickel-contaminated flowers compared with controls. Aluminum contamination did not appear to deter the bees. However, exposure to either metal may interfere with bees’ taste perception, agility, and working memory, all skills necessary for normal functioning. The results suggest that growing plants to bioremediate contaminated soil needs to be further examined with respect to its impact on pollinator populations.

**Figure:**
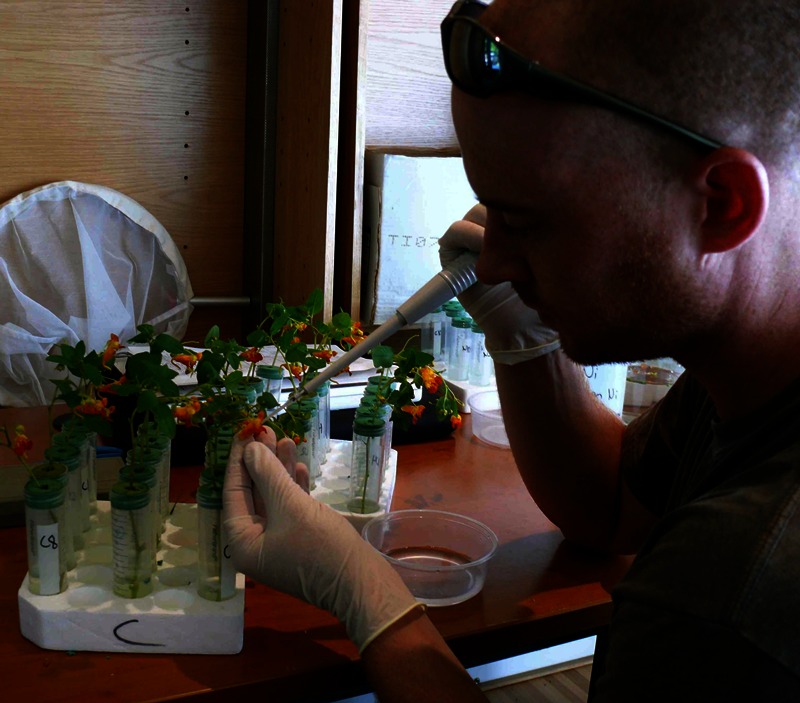
Investigators spiked jewelweed flowers with aluminum and nickel to test the effect on bumblebee behavior. University of Pittsburgh

## Magnetic Fields May Confound Cell Culture Studies

A survey of carbon dioxide tissue culture incubators found that ambient static and time-varying magnetic fields in six popular models differed by orders of magnitude between incubators as well as between locations centimeters apart within single units.^2^ Nearly 40% of points tested fell outside the normal geomagnetic field range. Fans and resistive heaters appeared to be the source of the extreme time-varying fields, while permanent magnets affected static fields. The authors argue that magnetic fields should be added to the list of variables that can influence cell biology experiments.

## EPA Announces the TRI University Challenge

The U.S. EPA is accepting applications until 13 May 2013 for a new program designed to discover innovative ways to utilize Toxics Release Inventory (TRI) data.^3^ The TRI is a database of emissions data submitted yearly by thousands of manufacturers. The TRI University Challenge is a bid by the EPA to increase the knowledge, use, and understanding of this repository of data. Priority will be given to programs related to pollution prevention and sustainability, stakeholder engagement, new uses for technology and data, and environmental education.

## Interaction between Cockroach Allergy and Air Pollution Exposure

Cockroach allergens are a major contributor to asthma in children, especially in lower-income urban environments. Investigators now report that children with higher prenatal exposure to both cockroach allergens and polycyclic aromatic hydrocarbons (PAHs) had an increased risk of developing cockroach allergy at ages 5 to 7 years.^4^ Children with a common mutation of the *GSTM* gene, which is involved in detoxification of PAHs, appeared particularly vulnerable. The findings suggest that reducing exposures to either allergens or combustion sources of PAHs could reduce asthma risk in urban communities.

**Figure:**
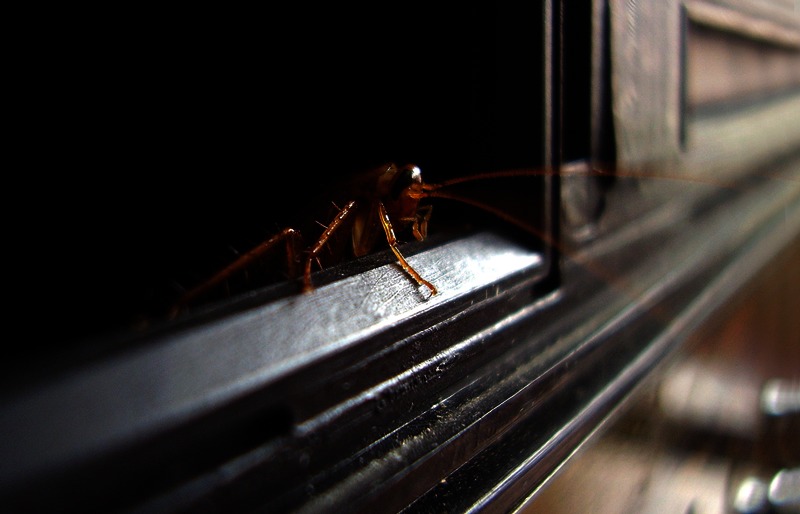
Cockroach allergens are an important trigger for asthma, especially in low-income urban populations. Vadim Kozlovsky/Shutterstock.com

## Washington Legislature Passes Asbestos-Labeling Bill

In March 2013 the Washington State Legislature passed a bill requiring that all asbestos-containing building materials sold in the state be clearly labeled.^5^ Asbestos is a known human carcinogen and is currently banned in dozens of countries, although not the United States.^6^ The fibrous material can be found in many building materials on the U.S. market, including cement siding, roofing materials, wallboard, spackle, and duct insulation.^7^ The sponsors of the Washington bill hope the labeling will promote informed consumer decision making. If the bill is signed by Governor Jay Inslee, the labeling requirements would take effect 1 January 2014.
